# Could *Dirofilaria immitis* contribute to occult allergic sensitization? Serological evidence from allergic patients in a hyperendemic area

**DOI:** 10.3389/falgy.2026.1943450

**Published:** 2026-09-29

**Authors:** José Alberto Montoya-Alonso, Alfonso Balmori-de la Puente, Iván Rodríguez-Escolar, Manuel Collado-Cuadrado, Alberto Gil-Abad, Elena Carretón, Rodrigo Morchón

**Affiliations:** 1 Research Institute of Biomedical and Health Sciences (IUIBS), University of Las Palmas de Gran Canaria, Las Palmas de Gran Canaria, Spain; 2 Zoonotic Diseases and One Health Group, Faculty of Pharmacy, Centre for Environmental Studies and Rural Dynamization (CEADIR), University of Salamanca, Salamanca, Spain; 3 Department of Ecology and Evolution, Doñana Biological Station, Seville, Spain; 4 Biomedical Research Institute of Salamanca (IBSAL), University of Salamanca, Salamanca, Spain

**Keywords:** allergic sensitization, *Dirofilaria immitis*, eosinophilia, human dirofilariosis, IgE, One Health

## Abstract

**Introduction:**

Although human infection with *Dirofilaria immitis* is classically linked to benign pulmonary nodules, the immunological impact of repeated exposure to infective larvae in allergic individuals remains poorly understood. This study evaluated serological evidence of exposure and sensitization to *D. immitis* in human patients with allergic diseases living in Gran Canaria, a hyperendemic area for heartworm disease.

**Methods:**

A total of 125 anonymized serum samples from patients diagnosed with allergic conditions were analyzed. Specific Immunoglobulin (Ig) G antibodies against *D. immitis* and *Wolbachia* sp., and specific IgE antibodies against D. immitis, were detected by enzyme-linked immunosorbent assay (ELISA). Associations between serological status, epidemiological variables, allergen sensitization, and eosinophilia were explored.

**Results:**

Anti-D. immitis IgE positivity was detected in 23.2% of patients, while 6.4% showed simultaneous IgG positivity against *D. immitis* and *Wolbachia*. Peripheral eosinophilia was significantly associated with anti-*D. immitis* IgE seropositivity, with a higher frequency of positives among patients with eosinophilia ≥6%.

**Discussion:**

These findings suggest that exposure to *D. immitis* may have immunological relevance in allergic patients from hyperendemic areas and support further investigation into its potential role in occult allergic sensitization.

## 1 Introduction

Dirofilariosis, caused by the nematode *Dirofilaria immitis*, is a vector-borne zoonotic disease primarily affecting domestic and wild canids, which act as definitive reservoirs. Over the last few decades, the geographical distribution of *D. immitis* has expanded across Europe, driven by climate change, environmental modification, increased vector suitability, and the spread of competent mosquito species. As a result, dirofilariosis is increasingly recognized as an emerging zoonotic disease with relevance for both veterinary and human medicine (1–3).

Humans are accidental and dead-end hosts for *D. immitis*. In most cases, the parasite does not reach sexual maturity, and human infection is classically associated with the development of benign pulmonary nodules or, less frequently, subcutaneous lesions (4). Diagnosis is usually based on imaging findings, histopathology, and/or serological evidence of exposure (4). However, the immunological consequences of repeated exposure to infective larvae in endemic areas remain insufficiently understood, particularly in individuals with pre-existing allergic disease or Th2-biased immune responses.

A crucial component in the pathogenesis of human pulmonary dirofilariosis is the presence of *Wolbachia pipientis*, an obligate endosymbiotic bacterium present in several filarial nematodes, including *D. immitis*. Host inflammatory responses are not directed exclusively against nematode antigens, but also against *Wolbachia* surface proteins, which have been associated with neutrophil activation, inflammatory mediator release, and specific IgG responses in humans (2, 5–9). Therefore, the detection of antibodies against both *D. immitis* and *Wolbachia* sp. may provide useful information on human exposure to infective larvae in endemic areas.

Allergic diseases are among the most common chronic conditions worldwide and affect a substantial proportion of the adult and pediatric population. Their prevalence has increased in recent decades, partly in association with environmental changes linked to climate change, air pollution, aeroallergen burden, and lifestyle-related factors. The most frequently reported allergic diseases include asthma, allergic rhinitis, and atopic dermatitis. Although sensitization to house dust mites, pollens, animal epithelia, and fungi explains many cases, the origin or exacerbating factors of allergic disease remain unclear in a proportion of patients (10, 11).

There is growing interest in the role of helminths as modulators or triggers of allergic inflammation. Helminth infections can induce strong type 2 immune responses characterized by eosinophilia, IgE production, mast cell activation, and cytokine profiles dominated by interleukin (IL) -4, IL-5, and IL-13. Depending on the parasite species, intensity and chronicity of exposure, and host immune background, helminths may either modulate allergic inflammation or contribute to allergic sensitization and symptom exacerbation (12). There is a growing number of reported associations between allergic manifestations and exposure to several helminths, including *Anisakis* spp., *Toxocara canis*, *Trichinella spiralis*, *Trichuris trichiura*, and *Trichuris suis* (13–16).

The Canary Islands provide a particularly suitable setting to investigate this issue. Gran Canaria is considered a hyperendemic area for *D. immitis*, with sustained transmission in animal reservoirs and favorable ecological conditions for mosquito vectors (9, 17). At the same time, the Canary Islands show a high prevalence of allergic diseases, including asthma (18.4%), rhinitis (40.3%), and atopic dermatitis (35.8%), in both adults and children (18–21). Although this high allergic burden has been partially attributed to sensitization to common environmental allergens, African dust intrusions, pollen exposure, and climatic factors, additional contributors may remain unidentified.

Previous studies have suggested that exposure to *D. immitis* may be associated with allergic sensitization in humans living in endemic areas. In particular, specific IgE responses against *D. immitis* have been detected in human populations from regions with high canine and feline infection rates, supporting the hypothesis that repeated exposure to infective larvae or parasite-derived antigens could contribute to occult allergic sensitization in susceptible individuals (17, 22). Nevertheless, evidence remains limited, and the relationship between *D. immitis* exposure, eosinophilia, and allergic disease has not been sufficiently characterized.

Therefore, the aim of the present study was to evaluate the serological evidence of exposure and IgE seroreactivity to *D. immitis* somatic antigens in patients diagnosed with allergic diseases living in Gran Canaria, a hyperendemic region for heartworm disease. Specifically, we assessed specific IgG responses against *D. immitis* and *Wolbachia*, specific IgE responses against *D. immitis*, and their association with epidemiological and clinical variables, with particular attention to peripheral eosinophilia.

## 2 Materials

### 2.1 Ethics statement

The present study was approved by the Human Research Ethics Committee of the University of Las Palmas de Gran Canaria (CEIH-2025-40). The approval covered the retrospective use of anonymized serum samples obtained from diagnostic archives of patients evaluated for allergic conditions, together with the minimum associated clinical data required to address the study objectives. No additional sampling, intervention, or patient contact was performed. The study was conducted in accordance with current European and national regulations on biomedical research, data protection, and the ethical use of anonymized human biological samples.

### 2.2 Study population

A total of 125 anonymized human serum samples from residents of Gran Canaria Island (Canary Islands, Spain) were retrospectively selected from a diagnostic serum archive of patients evaluated for allergic conditions. Gran Canaria has been reported as a hyperendemic area for dirofilariosis caused by *D. immitis* (17).

The study population consisted of 59 males and 66 females. Regarding age distribution, 76 individuals were under 18 years of age, 30 were aged 18–45 years, and 19 were aged 46–65 years. All patients had been diagnosed with or clinically evaluated for allergic disease, including rhinitis (*n* = 58), asthma (*n* = 39), asthma/rhinitis (*n* = 13), food allergy (*n* = 6), non-specified allergic processes (*n* = 3), and other allergic conditions (*n* = 6), including atopic dermatitis, urticaria, or combined allergic presentations. All the sera included were screened for antibodies against the most common human nematode infections, such as *Toxocara* and *Enterobius*, and were confirmed to be negative.

The minimum clinical and epidemiological information required for the study objectives was anonymously recorded, including sex, age group, allergic diagnosis, reported allergen sensitization, and peripheral eosinophil status. Reported allergen sensitization was available in 105 patients, whereas no specific allergen trigger was recorded in 20 patients. Peripheral eosinophilia was categorized using a threshold of ≥6%, according to the reference threshold used by the diagnostic laboratory. Overall, 38 individuals showed eosinophilia, whereas 87 individuals had eosinophil values within the reference range. Eosinophil status was included because peripheral eosinophilia is a clinically relevant marker of allergic inflammation and helminth-associated type 2 immune responses.

### 2.3 Detection of specific IgG and IgE antibodies

All serum samples were analyzed following the methodology previously described with minor modifications (17, 22). Specific IgG antibodies against *D. immitis* somatic antigens and recombinant *Wolbachia* surface protein (rWSP) (23) were detected using non-commercial enzyme-linked immunosorbent assays (ELISA).

Briefly, 96-well microplates were coated with 0.8 µg of somatic antigen extract from adult *D. immitis* worms (DiSA). Serum samples were analyzed at a 1:100 dilution for the detection of anti-*D. immitis* IgG and at a 1:40 dilution for the detection of anti-rWSP IgG. A goat anti-human IgG (H + L) antibody conjugated to horseradish peroxidase (Thermo-Fisher, Barcelona, Spain) was used as the secondary antibody at a 1:4000 dilution. Optical densities (ODs) were measured at 492 nm using an Easy Reader microplate reader (Bio-Rad Laboratories, Hercules, CA, USA). The cut-off values were established at OD = 0.8 for anti-*D. immitis* IgG and OD = 0.5 for anti-rWSP IgG. Individuals were considered IgG-positive only when simultaneous positive reactions against both *D. immitis* somatic antigens and rWSP were detected.

Subsequently, specific IgE antibodies against *D. immitis* were evaluated using a non-commercial ELISA. Briefly, 96-well microplates were coated with 0.8 µg of DiSA. Serum samples were analyzed at a 1:100 dilution. A goat anti-human IgE (H + L) conjugated to horseradish peroxidase (Thermo Fisher Scientific, Barcelona, Spain) was used as the secondary antibody at a 1:100 dilution. Optical densities were measured at 492 nm using an Easy Reader microplate reader (Bio-Rad Laboratories, Hercules, CA, USA). The cut-off value for anti-*D. immitis* IgE positivity was established at OD = 0.6, calculated as the mean OD plus three standard deviations of 34 serum samples from clinically healthy blood donors living in a *D. immitis*-free area. All these samples tested negative for *Anisakis*, *Ascaris* and *Toxocara* (data not shown).

### 2.4 Statistical analysis

Statistical analyses were performed using R software (version 4.2.3). Descriptive analyses were first conducted to summarize the frequency distribution of serological results according to sex, age group, allergic diagnosis, reported IgE seroreactivity to *D. immitis* somatic antigens, and eosinophil status.

Binary logistic regression models were then used to explore the association between patient characteristics and serological status. The dependent variables were coded as negative/positive (0/1) and included: anti-*D. immitis*/anti-rWSP IgG status, anti-*D. immitis* IgE status, and combined IgG/IgE serological status. The explanatory variables evaluated were sex, age group, clinical diagnosis, reported IgE seroreactivity to *D. immitis* somatic antigens, and eosinophil status. Model selection was performed using a bidirectional stepwise procedure based on the Akaike Information Criterion (AIC), in order to identify the most parsimonious model.

Pairwise associations between variables retained in the selected models and the serological status were further evaluated using Fisher's exact test, as recommended for categorical data with small expected frequencies. Differences in seropositivity frequencies were visualized using stacked bar plots. Statistical significance was set at *p* < 0.05.

## 3 Results

### 3.1 Descriptive results

The serological results obtained from the 125 allergic patients are summarized in Table 1. Overall, 8/125 patients (6.4%) were positive for both anti-*D. immitis* IgG and anti-*Wolbachia* IgG. In contrast, 29/125 patients (23.2%) showed specific anti-*D. immitis* IgE positivity. Among IgE-positive patients, 8/29 (27.6%) were also positive for anti-*D. immitis* IgG and anti-*Wolbachia* IgG.

**Table 1:** Seroprevalence of anti-*dirofilaria immitis* igG/anti-*wolbachia* igG, anti-*D. immitis* IgE, and combined IgG/IgE positivity according to sex, age group, clinical diagnosis, reported allergen sensitization, and eosinophil status.

		IgG+ *D. immitis/Wolbachia*		IgE + *D. immitis*		IgG+ *D. immitis/Wolbachia* IgE + *D. immitis*	
Variable	**n**	**+**	**%**	**+**	**%**	**+**	**%**
Sex							
Male	59	3	5.08	12	20.34	3	5.08
Female	66	5	7.58	17	25.76	5	7.58
Age (years)							
<18	76	6	7.89	20	26.32	6	7.89
18–45	30	2	6.67	5	16.67	2	6.67
46–65	19	0	0.00	4	21.05	0	0.00
Diagnosis							
Rhinitis	58	3	5.17	15	25.86	3	5.17
Asthma	39	4	10.26	9	23.08	4	10.26
Allergic process	3	1	33.33	2	66.67	1	33.33
Asthma/Rhinitis	13	0	0.00	1	7.69	0	0.00
Food allergy	6	0	0.00	1	16.67	0	0.00
Atopic dermatitis	1	0	0.00	1	100.00	0	0.00
Drug allergy	1	0	0.00	0	0.00	0	0.00
COPD	1	0	0.00	0	0.00	0	0.00
Rhinitis/Atopic dermatitis	1	0	0.00	0	0.00	0	0.00
Rhinitis/Urticaria	1	0	0.00	0	0.00	0	0.00
Urticaria	1	0	0.00	0	0.00	0	0.00
Allergens							
Detected	105	8	7.62	27	25.71	8	7.62
Not reported/ detected	20	0	0.00	2	10.00	0	0.00
Eosinophils							
≥6% (eosinophilia)	38	3	7.89	16	42.11	3	7.89
<6% (within range)	87	5	5.75	13	14.94	5	5.75
TOTAL	125	8	6.40	29	23.20	8	6.40

*n*: number of patients; +: number of positive patients; %: percentage of positive patients within each category.

Anti-*D. immitis* IgE positivity was detected in both males and females, with 12/59 positive males (20.3%) and 17/66 positive females (25.8%). Combined IgG/IgE positivity was observed in 3/59 males (5.1%) and 5/66 females (7.6%).

When age groups were analyzed, the highest number of IgE-positive cases was observed in individuals under 18 years of age, with 20/76 positive patients (26.3%). In the 18–45-year age group, 5/30 patients (16.7%) were IgE-positive, whereas 4/19 patients (21.1%) were IgE-positive in the 46–65-year age group. Combined IgG/IgE positivity was detected in 6/76 individuals under 18 years of age (7.9%) and in 2/30 individuals aged 18–45 years (6.7%), whereas no combined IgG/IgE-positive cases were detected in the 46–65-year age group.

Regarding clinical diagnosis, most IgE-positive patients had rhinitis or asthma, which were also the most frequent clinical diagnoses in the study population. Anti-*D. immitis* IgE positivity was detected in 15/58 patients with rhinitis (25.9%) and in 9/39 patients with asthma (23.1%). Combined IgG/IgE positivity was observed in 3/58 patients with rhinitis (5.2%) and in 4/39 patients with asthma (10.3%). Positive cases were also detected in some less frequent diagnostic categories, although these findings should be interpreted descriptively due to the small number of patients in each subgroup. Specifically, anti-*D. immitis* IgE positivity was detected in 1/13 patients with asthma/rhinitis, 1/6 patients with food allergy, 2/3 patients with a non-specified allergic process, and 1/1 patient with atopic dermatitis. Among these less frequent categories, combined IgG/IgE positivity was observed only in one patient with a non-specified allergic process.

Among patients with reported allergen sensitization, 27/105 (25.7%) were IgE-positive and 8/105 (7.6%) showed combined IgG/IgE positivity. In contrast, among patients with no reported allergen sensitization, 2/20 (10.0%) were IgE-positive and none showed combined IgG/IgE positivity.

Peripheral eosinophilia was associated with a higher frequency of anti-*D. immitis* IgE positivity. Among patients with eosinophil values within the reference range, 13/87 (14.9%) were IgE-positive, whereas 16/38 patients with eosinophilia ≥6% (42.1%) were IgE-positive. Combined IgG/IgE positivity was observed in 5/87 patients without eosinophilia (5.7%) and in 3/38 patients with eosinophilia ≥6% (7.9%).

### 3.2 Regression models and association analyses

Logistic regression models were used to explore the association between patient characteristics and serological status. For anti-*D. immitis*/anti-*Wolbachia* IgG positivity, reported allergen sensitization was the only variable retained in the selected model, although without a non-significant risk estimation (OR < 0.001, *p* = 0.996). For anti-*D. immitis* IgE positivity, eosinophil status was retained as the most relevant predictor, with individuals presenting normal eosinophil counts displaying substantially lower odds of positivity compared to those with eosinophilia (OR = 0.28, 95% CI: 0.10–0.72, *p* = 0.009). For combined IgG/IgE serological status, reported allergen sensitization was the only variable retained in the selected model, without reliable risk estimation (OR < 0.001, *p* = 0.996).

Pairwise analyses using Fisher's exact test showed a significant association between eosinophilia and anti-*D. immitis* IgE positivity (*p* < 0.01), with a higher proportion of IgE-positive patients among those with eosinophilia ≥6% (Figure 1). No significant associations were observed between anti-*D. immitis* IgE positivity and sex, age group, clinical diagnosis, or reported allergen sensitization (*p* > 0.05).

**Figure 1:**
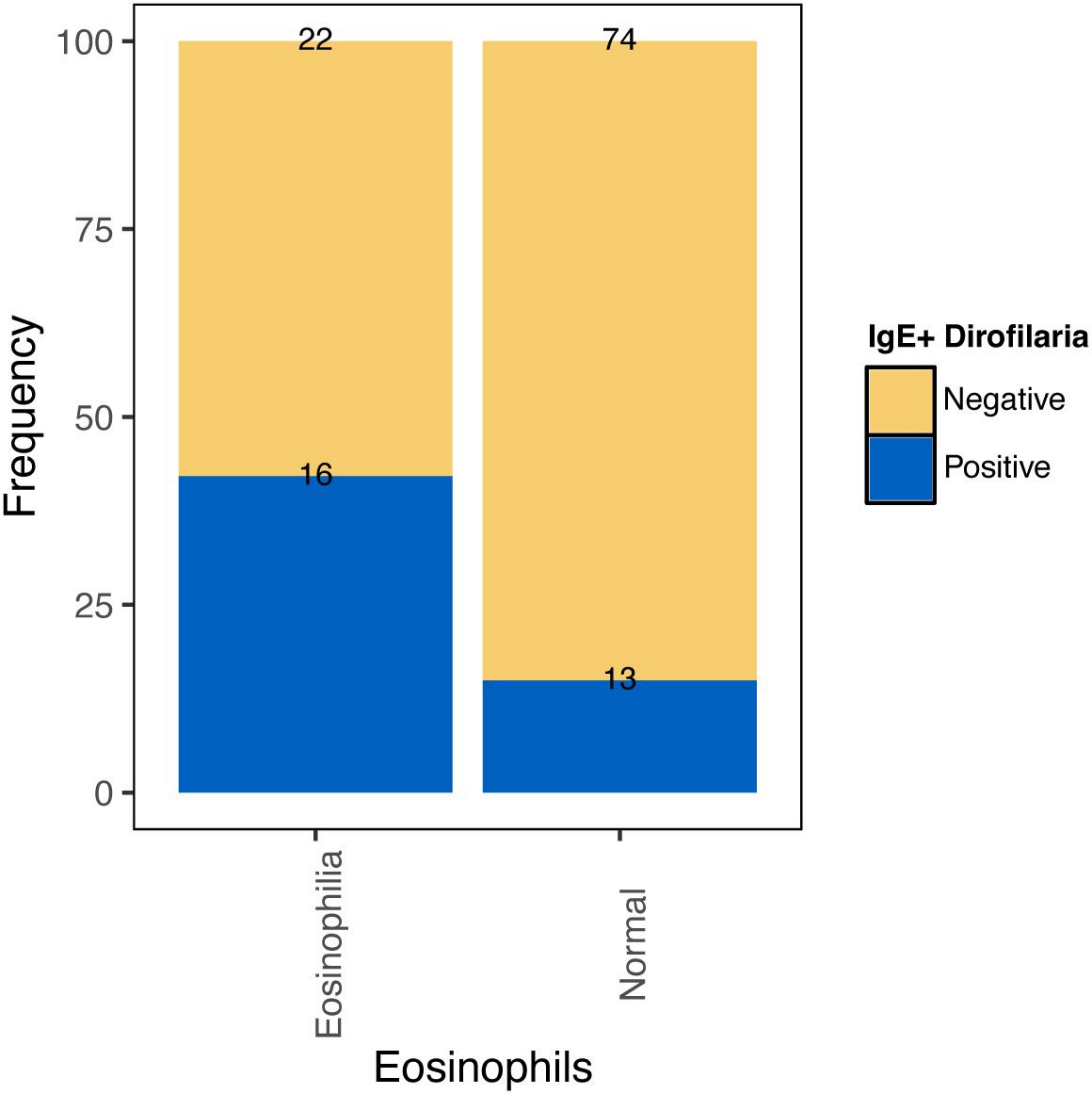
Distribution of anti-*Dirofilaria immitis* igE-positive and igE-negative patients according to eosinophil status. Bars represent relative frequencies, and numbers within the bars indicate the number of patients in each serological category. Anti-*D. immitis* IgE positivity was more frequent among patients with eosinophilia than among those with normal eosinophil levels.

All patients with anti-*D. immitis*/anti-*Wolbachia* IgG positivity, as well as those with combined IgG/IgE positivity, belonged to the group with reported allergen sensitization. However, Fisher's exact test did not show a statistically significant association between reported allergen sensitization and either IgG positivity or combined IgG/IgE positivity (*p* = 0.35). No significant associations were observed between IgG positivity or combined IgG/IgE positivity and sex, age group, clinical diagnosis, or eosinophil status (*p* > 0.05) (Figure 2).

**Figure 2:**
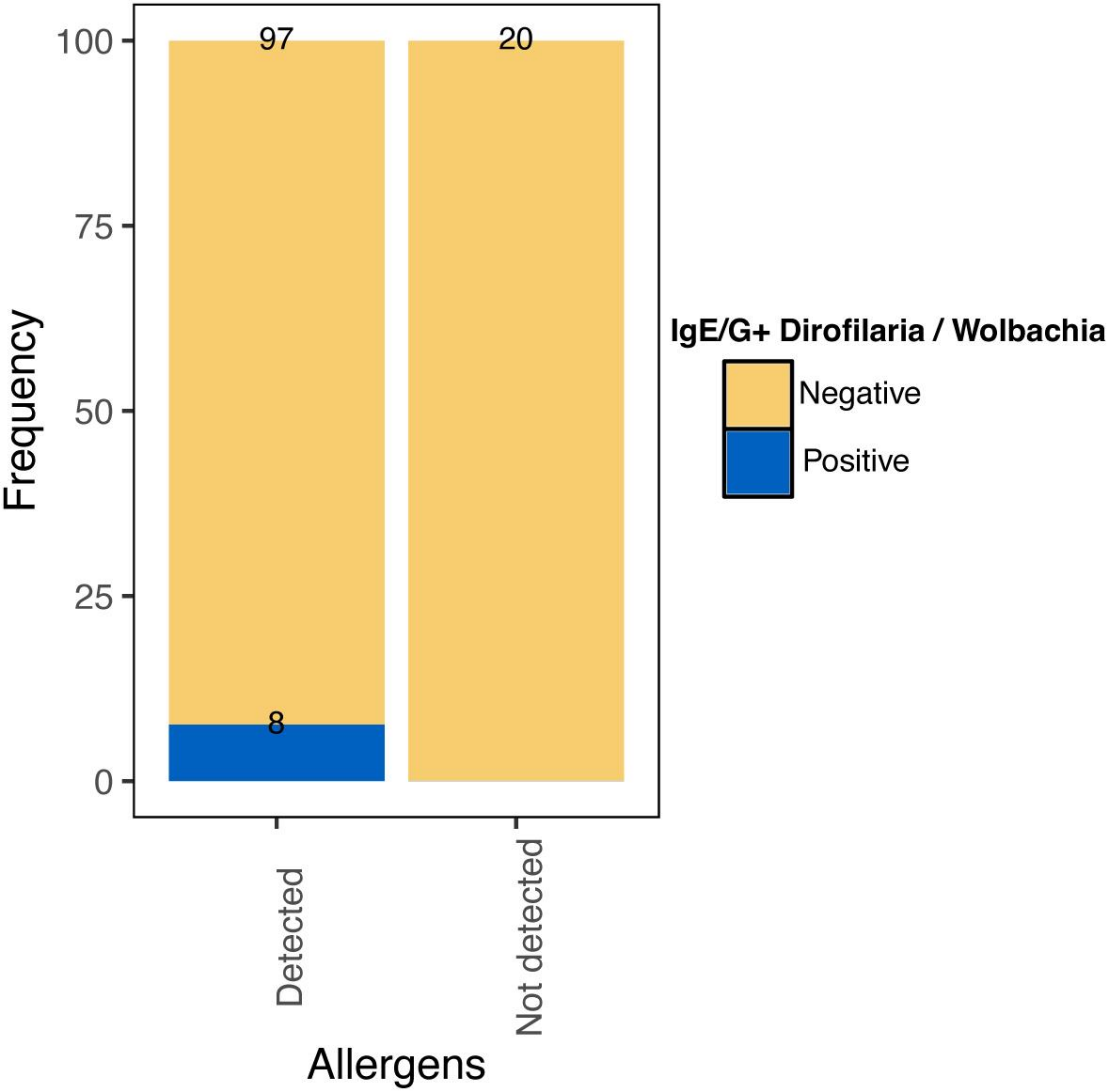
Distribution of anti-*Dirofilaria immitis* igE and anti-*D. immitis*/anti-*Wolbachia* IgG serological status according to reported allergen sensitization. Bars represent relative frequencies of patients with detected and non-detected allergen sensitization. Numbers within the bars indicate the number of patients in each serological category.

## 4 Discussion

The present study provides new evidence supporting the hypothesis that exposure to *Dirofilaria immitis* may be associated with allergic sensitization in humans living in hyperendemic areas such as Gran Canaria Island (17). In this cohort of patients with allergic diseases, the seroprevalence of specific anti-*D. immitis* IgE was 23.2%, which is higher than the seroprevalence previously reported in the general population from the same region (8.27%) (22). Although the cross-sectional design of the present study does not allow causal relationships to be established, this finding supports the need to further investigate *D. immitis* exposure as a potential environmental and vector-borne contributor to allergic sensitization in endemic regions.

From a *One Health* perspective, these results are relevant because they link a vector-borne zoonotic parasite, animal reservoirs, mosquito exposure, and human immune responses within the same ecological context. Gran Canaria represents a particularly suitable scenario for this type of investigation, given the high endemicity of *D. immitis* in dogs and cats and the high burden of allergic disease reported in the human population (17, 22). In this scenario, repeated exposure to infective larvae transmitted by mosquito vectors could act as a source of parasite-derived antigens capable of stimulating or modulating immune responses in susceptible individuals.

A relevant finding of this study was the detection of specific IgG antibodies against *D. immitis* and *Wolbachia* sp. in 6.4% of patients. Humans are accidental and non-permissive hosts for *D. immitis*, and the parasite rarely completes its life cycle. Therefore, serological evidence of exposure to both parasite antigens and *Wolbachia* sp. may reflect contact with infective larvae and subsequent immune recognition of nematode and endosymbiotic bacterial components (24, 25). *Wolbachia* sp. has been traditionally associated with inflammatory responses in filarial infections, including neutrophil activation, IgG responses, and thromboxane B_2_ production in human pulmonary dirofilariosis (5, 26). In the context of allergic disease, this exposure may contribute to a broader inflammatory environment, although the specific contribution of *Wolbachia* sp. to type 2 immune polarization requires further investigation.

The most consistent statistical finding in the present study was the association between peripheral eosinophilia and anti-*D. immitis* IgE seropositivity. Patients with eosinophilia ≥6% showed a higher frequency of specific IgE positivity than patients with eosinophil values within the reference range. This result is biologically plausible, as eosinophilia is a common feature of both allergic inflammation and helminth-associated type 2 immune responses, which may maintain a state of persistent eosinophilic activation (27). However, the direction of this association cannot be determined from the present data. It is possible that patients with a pre-existing atopic background are more prone to develop IgE responses against parasite antigens, or alternatively, that repeated exposure to *D. immitis* antigens may contribute to maintaining eosinophilic inflammation in sensitized individuals.

Similar associations between helminth exposure, IgE responses, eosinophilia, and respiratory allergic disease have been reported for other zoonotic or soil-transmitted helminths, including *Toxocara canis* and *Ascaris lumbricoides* for which exposure or infection has been linked to increased asthma severity and bronchial hyperreactivity (27–29). In the present cohort, rhinitis and asthma were the most frequent diagnoses among IgE-positive patients, although these were also the most common clinical diagnoses in the study population. Therefore, these findings should not be interpreted as evidence of a diagnosis-specific association, but rather as an indication that IgE seroreactivity to *D. immitis* somatic antigens may occur in patients with common respiratory allergic phenotypes living in endemic areas (22).

The relationship between helminth exposure and allergy is complex. According to the hygiene hypothesis, some chronic helminth infections may exert immunomodulatory effects and reduce allergic inflammation (30). However, this effect is not universal and depends on several factors, including parasite species, infection intensity, chronicity of exposure, host immune status, and environmental context (12, 30, 31). In the case of *D. immitis*, humans are not permissive hosts, and exposure is likely to consist of repeated inoculation of larvae or parasite-derived antigens in endemic areas, without establishment of patent infection. In such a scenario, exposure may be insufficient to induce regulatory mechanisms associated with chronic helminth infection, but sufficient to promote antigenic stimulation in predisposed individuals. This hypothesis is consistent with the higher frequency of anti-*D. immitis* IgE observed in allergic patients, but it should be confirmed in controlled longitudinal studies.

The absence of significant associations between serological status and sex, age group, or clinical diagnosis suggests that exposure may be broadly distributed across the studied population. Although the highest number of IgE-positive patients was observed among individuals under 18 years of age, age was not significantly associated with anti-*D. immitis* IgE positivity. Therefore, the apparent predominance of pediatric cases should be interpreted cautiously, particularly because individuals under 18 years of age represented the largest age group in the study population. Nevertheless, age-related differences in outdoor exposure, mosquito contact, or the tendency of younger individuals with allergic disease to develop IgE-mediated responses could also contribute to this pattern (26, 27). Future studies including age-matched control groups would be necessary to determine whether children are truly at higher risk of sensitization.

Several limitations should be acknowledged. First, anti-*D. immitis* IgE was evaluated using a crude adult somatic antigen extract (DiSA) rather than recombinant or purified *D. immitis*-specific allergens, as the latter are not currently available. Consequently, this prevents the identification of the specific allergenic molecules driving the response. Nevertheless, specific exposure to *D. immitis* is strongly supported by the co-positivity for anti-*Wolbachia* IgG antibodies in our study population, particularly considering that Gran Canaria is an island free of autochthonous or endemic lymphatic filariae. Second, the cross-sectional design prevents any causal inference between *D. immitis* exposure and allergic disease. Third, a major limitation is the absence of a contemporaneous non-allergic control group, as comparisons with the general population rely on previously published data. This limits the ability to determine whether seropositivity is truly associated with allergic disease or merely reflects background exposure in the general population. Fourth, as with other nematode serological assays, cross-reactivity cannot be completely excluded. The lack of specific cross-reactivity testing with other helminths and invertebrates means there is a possibility that the detected IgE responses are not entirely specific to *D. immitis*. This is particularly relevant in all IgG/IgE-positive patients, who also showed sensitization to other common allergens (29, 32). The biological explanation of cross-reactivity between invertebrate pan-allergens, such as tropomyosins shared by mites, nematodes, and other invertebrates, or a summation effect, in which the parasitic burden lowers the tolerance threshold to other environmental allergens, cannot be ruled out. Although the detection of antibodies against *Wolbachia* sp. may increase the specificity of exposure assessment (5, 33), it does not fully eliminate the possibility of cross-reactive responses. Fifth, the study lacks direct proof of active infection (e.g., via imaging techniques, histopathology, or molecular methods); therefore, seropositivity serves as an indicator of immunological exposure to the agent rather than confirming an active infection itself. Sixth, detailed epidemiological information regarding exposure (e.g., frequency of mosquito bites, proximity to reservoir hosts, and use of repellents) was not obtained, making it impossible to adequately evaluate specific risk factors. Finally, the number of patients positive for both anti-*D. immitis* IgG and anti-*Wolbachia* IgG was low, limiting the statistical power to detect associations with epidemiological or clinical variables.

Despite these limitations, the present findings suggest that *D. immitis* exposure should be considered as a potential contributor to IgE seroreactivity to *D. immitis* somatic antigens in hyperendemic regions. The association between anti-*D. immitis* IgE positivity and eosinophilia supports the biological plausibility of this relationship and highlights the need for further studies integrating parasitological, immunological, clinical, and environmental data. Future research should include non-allergic control groups, longitudinal designs, molecular allergen characterization, and assessment of potential cross-reactivity with common environmental allergens.

In conclusion, although causality cannot be established due to the cross-sectional nature of the study, the IgE/IgG serologigal reactivity to *D. immitis* somatic antigens observed in allergic patients, together with its significant association with peripheral eosinophilia, suggests that exposure to this parasite may have immunological relevance in humans living in hyperendemic areas. In the context of climate change, vector expansion, and sustained transmission in animal reservoirs, human dirofilariosis should not be viewed exclusively as a rare pulmonary disease, but also as a potential contributor to occult allergic sensitization in endemic settings. Further controlled and longitudinal studies are needed to clarify the causal relevance of these findings, characterize potential cross-reactive mechanisms, and better define the parasite–host immune interactions involved in allergic responses.

## Data Availability

The raw data supporting the conclusions of this article will be made available by the authors, without undue reservation.
